# Activation of the NF-κB pathway as a mechanism of alcohol enhanced progression and metastasis of human hepatocellular carcinoma

**DOI:** 10.1186/s12943-014-0274-0

**Published:** 2015-01-27

**Authors:** Fei Wang, Jin-Lian Yang, Ke-ke Yu, Mei Xu, You-zhi Xu, Li Chen, Yan-min Lu, Hao-shu Fang, Xin-yi Wang, Zhong-qian Hu, Fei-fei Li, Lixin Kan, Jia Luo, Si-Ying Wang

**Affiliations:** Department of Pathophysiology, School of Basic Medical Science, Anhui Medical University, 81 MeiShan Road, Hefei, Anhui 230032 P.R. China; Department of Pharmacology and Nutritional Sciences, University of Kentucky College of Medicine, Lexington, Kentucky 40536 USA; Department of Burns, The First Affiliated Hospital of Anhui Medical University, Hefei, Anhui 230022 P.R. China; Department of Clinical Medicine, Anhui Medical University, Hefei, Anhui 230032 P.R. China

**Keywords:** Alcohol, Angiogenesis, Human hepatocellular cancer, Metastasis, Reactive oxygen species

## Abstract

**Background:**

Hepatocellular carcinoma (HCC), the most common form of primary liver cancer, is the third leading cause of cancer-related death in human. Alcohol is a known risk factor for HCC. However it is still unclear whether and how alcohol enhances the progression and metastasis of existing HCC.

**Methods and results:**

We first retrospectively investigated 52 HCC patients (24 alcohol-drinkers and 28 non-drinkers), and found a positive correlation between alcohol consumption and advanced Tumor-Node-Metastasis (TNM) stages, higher vessel invasion and poorer prognosis. *In vitro* and *in vivo* experiments further indicated that alcohol promoted the progression and migration/invasion of HCC. Specifically, in a 3-D tumor/endothelial co-culture system, we found that alcohol enhanced the migration/invasion of HepG2 cells and increased tumor angiogenesis. Consistently, higher expression of VEGF, MCP-1 and NF-κB was observed in HCC tissues of alcohol-drinkers. Alcohol induced the accumulation of intracellular reactive oxygen species (ROS) and the activation of NF-κB signaling in HepG2 cells. Conversely, blockage of alcohol-mediated ROS accumulation and NF-κB signaling inhibited alcohol-induced expression of VEGF and MCP-1, the tumor growth, angiogenesis and metastasis.

**Conclusion:**

This study suggested that chronic moderate alcohol consumption may promote the progression and metastasis of HCC; the oncogenic effect may be at least partially mediated by the ROS accumulation and NF-ĸB-dependent VEGF and MCP-1 up-regulation.

## Background

Hepatocellular carcinoma (HCC) is the most common primary liver cancer and accounts for up to 70-85% of primary liver cancers [1]. Advanced HCC carries a poor prognosis with a five-year survival rate <10% [2]. It is well recognized that both genetic and environmental factors contribute to human HCC. Alcohol, an important environmental factor, was classified as a group I carcinogen and chronic alcohol consumption has been recognized as an important risk factor for liver cancers [3,4]. In addition to act as risk factor for carcinogenesis, epidemiological studies also indicated that alcohol consumption is often associated with advanced and aggressive tumors [5]. Although alcohol is clearly recognized as a risk factor for liver cancers, its role in cancer progression and metastasis is currently unknown. We specifically hypothesized that chronic alcohol consumption might enhance HCC progression and metastasis.

Tumor angiogenesis, the formation of new blood vessels from endothelial precursors within tumors, is a prerequisite step for growth and progression of solid malignancies [6]. Vascular endothelial growth factor (VEGF) is an essential player in tumor angiogenesis and mediates tumor aggressiveness [7-9]. Monocyte chemotactic protein-1 (MCP-1), a key CC chemokine responsible for trafficking and activation of monocytes/macrophages has been recognized as an important angiogenic chemokine [10,11]; it also plays a critical role in solid tumors [12]. Previous studies indicated that alcohol exposure up-regulated the expression of VEGF and MCP-1 in other experimental models [13]. It has also been reported that the expression of VEGF and MCP-1 was regulated by reactive oxygen species and NF-ĸB [14,15]. Based on these findings, we further hypothesized that chronic alcohol consumption might enhance HCC progression and metastasis through up-regulating the expression of VEGF/MCP-1, which, in turn, may be regulated by reactive oxygen species and the NF-ĸB signaling pathway. We tested this hypothesis by retrospectively analyzing clinical data, and functional analysis using experimental models.

## Results

### Alcohol consumption is associated with advanced TNM stages, greater vessel invasion and poorer prognosis

We retrospectively surveyed 52 HCC patients, among which 24 patients consumed alcohol during last 10–40 years and 28 patients were no-drinkers. No statistical differences were found in the age, hepatitis B virus (HBV) infection, presence of cirrhosis, liver function (Child grade), tumor size, or number of tumors between the alcoholic and nonalcoholic patients (*P* >0.05), except for gender (*P* =0.026; Table 1). We categorized the alcohol drinkers into two groups, i.e., low (<35 g/day) and moderate-heavy consumption (35–87.5 g/day and ≥ 87.5 g/day) based on the average number of drinks they stated. We calculated the amount of ethanol ingested by assuming 35 g of ethanol per 100 ml of about 43% v/v spirits. We used the Tumor-Node-Metastasis (TNM) of International Union Against Cancer (UICC) system to further classify the stages of the HCC. Specifically, UICC TNM is comprised of 4 stages (I, II, III, IV) based on growth pattern (single or multiple), size (≤5 cm or >5 cm), vascular invasion, and extrahepatic spread. We further analyzed the effects of alcohol intake on tumor progression and examined the relationship between the amount alcohol consumption and the extent of tumor progression. According to the multivariate binary logistic regression analysis adjusted for sex, age, HBV infection and liver function (Child grade), the moderate and high drinkers(35–87.5 g/day and ≥ 87.5 g/day) had a significantly higher percentage of advanced tumors (TNM stage III-IV) (OR =22.99; 95% CI: 2.13-248.91; *P* =0.010), and higher vessel invasion (OR =21.04; 95% CI: 1.61-275.56, *P* =0.020) (Table 2). There was no significant correlation with tumor differentiation (data not shown). However, patients with low alcohol consumption (<35 g/day) and nondrinkers did not display statistical difference in TNM stage and vessel invasion.

**Table 1:** **Univariate analysis of tumor characteristics and alcohol consumption in the 52 HCC patients**

**Variables**		**Never**	**Alcohol consumption n (%)**		
			**Ever**	**χ** ^**2**^	***P*** **value**
Sex	Male	21(46.7)	24(53.3)	4.953	0.026^a^
	Female	7(100)	0(0)		
Age(yr)	≤50	19(59.4)	13(40.6)	1.023	0.312
	>50	9(45)	11(55)		
HBV infection	Negative	4(45.4)	5(55.6)	0.065	0.799
	Positive	24(55.8)	19(44.2)		
Cirrhosis	Negative	7(53.8)	6(46.2)	0.000	1.000
	Positive	21(53.8)	18(46.2)		
Child grade	A	26(54.2)	22(45.8)	__	0.505^*^
	B	1(33.3)	2(66.7)		
	C	1(100)	0(0)		
Tumor size(cm)	≤5	15(51.7)	14(48.3)	0.119	0.730
	>5	13(56.5)	10(43.5)		
Number of tumors	1	27(55.1)	22(44.9)	0.119	0.730
	≥2	1(33.3)	2(66.7)		

^a^ *P* < 0.05; ^*^exact *P.*

**Table 2:** **Odds ratios of TNM stage and vessel invasiveness in relation to different levels of alcohol consumption**

	**TNM I-II**		**TNM III-IV**					
	**Control(n = 39)**		**Cases(n = 13)**		**Odds ratio** ^*^	**95% confidence interval**		***P*** **-Value**
	**NO.**	**%**	**NO.**	**%**				
Average ethanol intake								
0	25	64.2	3	23.1	1.00^†^			x
Low(<35 g/d)	7	17.9	2	15.4	5.81	0.42	80.57	0.190
Moderate(≥35 < 87.5 g/d) & High(≥87.5 g/d)	7	17.9	8	61.5	22.99	2.13	248.91	0.010^a^
Vessel invasiveness								
	**Control(n = 43)**		**Cases(n = 9)**		**Odds ratio** ^*^	**95% confidence interval**		***P*** **-Value**
	**NO.**	**%**	**NO.**	**%**				
Average ethanol intake								
0	27	62.8	1	11.1	1.00^†^			
Low(<35 g/d)	8	18.6	1	11.1	2.05	0.09	46.40	0.653
Moderate(≥35 < 87.5 g/d) & High(≥87.5 g/d)	8	18.6	7	77.8	21.04	1.61	275.56	0.020^a^

^*^Adjusted for sex, age, HBV infection and liver function (Child grade).

^†^Reference category.

^a^ *P* < 0.05.

**Figure 1:**
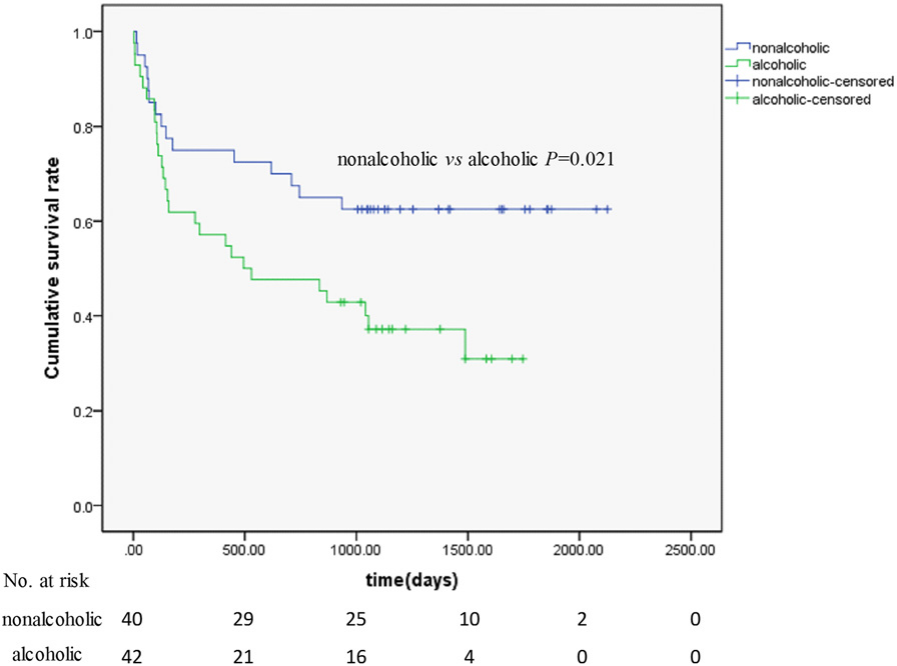
**Effects of alcohol consumption on the prognosis of patients with hepatocellular cancer.** The overall survival time in 82 HCC patients (40 none-drinkers and 42 alcohol drinkers) during the period of 2006–2009 was analyzed and presented with Kaplan-Meier curves. Mean survival time was 27.4 months for non-drinkers and 47.8 months for drinkers, respectively. Alcohol consumption significantly shorted life span of patients (*P* = 0.021). The number of patients at risk was listed below the survival curves.

To further clarify this issue, we included more eligible HCC patients for the evaluation of correlation between alcohol consumption and the prognosis. Eighty-two HCC patients, including 52 cases in first cohort which were confirmed by post-operative pathology from 2006 to 2009, were followed-up for 70 months. Clinical characteristics of the 82 HCC patients were summarized in Table 3. Forty of them were nondrinkers and forty two are alcohol users. At the end of the following-up, 42 patients died. Three alcoholic and 2 nonalcoholic patients died of liver failure after surgery. Twenty-four alcoholic and 13 nonalcoholic patients die due to tumor recurrence and metastasis. According to the multivariate Cox regression analysis, the TNM stage, liver function (Child grade C) and alcohol consumption were identified as independent prognostic factors for death due to HCC recurrence. It also showed that the hazard ratio of alcoholic versus nonalcoholic patients was 4.576 (95% CI: 1.854-11.293, *P* < 0.01) (Table 4). The overall survival rates for nondrinking HCC patients at 12 and 36 months were 75% and 62.5% respectively; however, the same rates for alcohol drinking HCC patients were 57.15% and 35.7% respectively. The mortality at 70 months in alcoholic patients was significantly higher than that of nonalcoholic patients [64.3% (27/42) *vs* 37.5% (15/40), *P* =0.015]. Alcohol consumption significantly shorted life span of patients (*P* =0.021) (Figure 1). Thus, HCC patients who were alcohol drinkers were associated with advanced TNM stages, higher vessel invasion and poorer prognosis.

**Table 3:** **Major clinical characteristics of the 82 HCC patients**

**Covariate**		**All cases (n = 82)**
Sex	Male, n (%)	67(81.7)
	Female	15(18.3)
Age (yr)	Mean ± SD	50.9 ± 13.1
Alcohol history	Never, n (%)	40(48.8)
	Ever	42(51.2)
HBV infection	Negative, n (%)	11(13.4)
	Positive	71(86.6)
Maximal tumor size(cm)	Mean ± SD	6.33 ± 4.03
Tumor grade	Well differentiated, n (%)	18(22.0)
	Moderately differentiated	51(62.2)
	Poorly differentiated	13(15.9)
TNM stage	I-II, n (%)	50(61.0)
	III-IV	32(39.0)
Vessel invasiveness	Negative, n (%)	64(78.0)
	Positive	18(22.0)
Child grade	A, n (%)	64(78.0)
	B	14(17.1)
	C	4(4.9)
Cirrhosis	Negative, n (%)	21(25.6)
	Positive	61(74.4)

**Table 4:** **Cox regression analysis of risk factors for death due to recurrence in 82 HCC patients**

**Variables**	**HR**	**95 % confidence interval**		***P-*** **value**
Sex	2.469	0.850	7.173	0.097
Age	0.863	0.430	1.732	0.679
HBV infection	0.665	0.250	1.767	0.413
TNM stage	4.089	1.864	8.972	0.000^b^
Liver function(Child grade) A		0.038		
B	2.092	0.846	5.174	0.110
C	4.185	1.162	15.074	0.029^a^
Alcohol consumption	4.576	1.854	11.293	0.001^b^

HR: Hazard ratio; Sex: female *vs* male (HR: 2.469); Age: >50 yr *vs* ≤50 (HR: 0.863); HBV infection: positive *vs* negative (HR: 0.665); TNM: III-IV *vs* I-II (HR: 4.089); Liver function (Child grade): B *vs* A (HR: 2.092), C *vs* A (HR: 4.185); Alcohol consumption: alcoholic *vs* nonalcoholic (HR: 4.576); ^a^ *P* < 0.05; ^b^ *P* < 0.01.

### Alcohol consumption is associated with enhanced angiogenesis in HCC tissues

**Figure 2:**
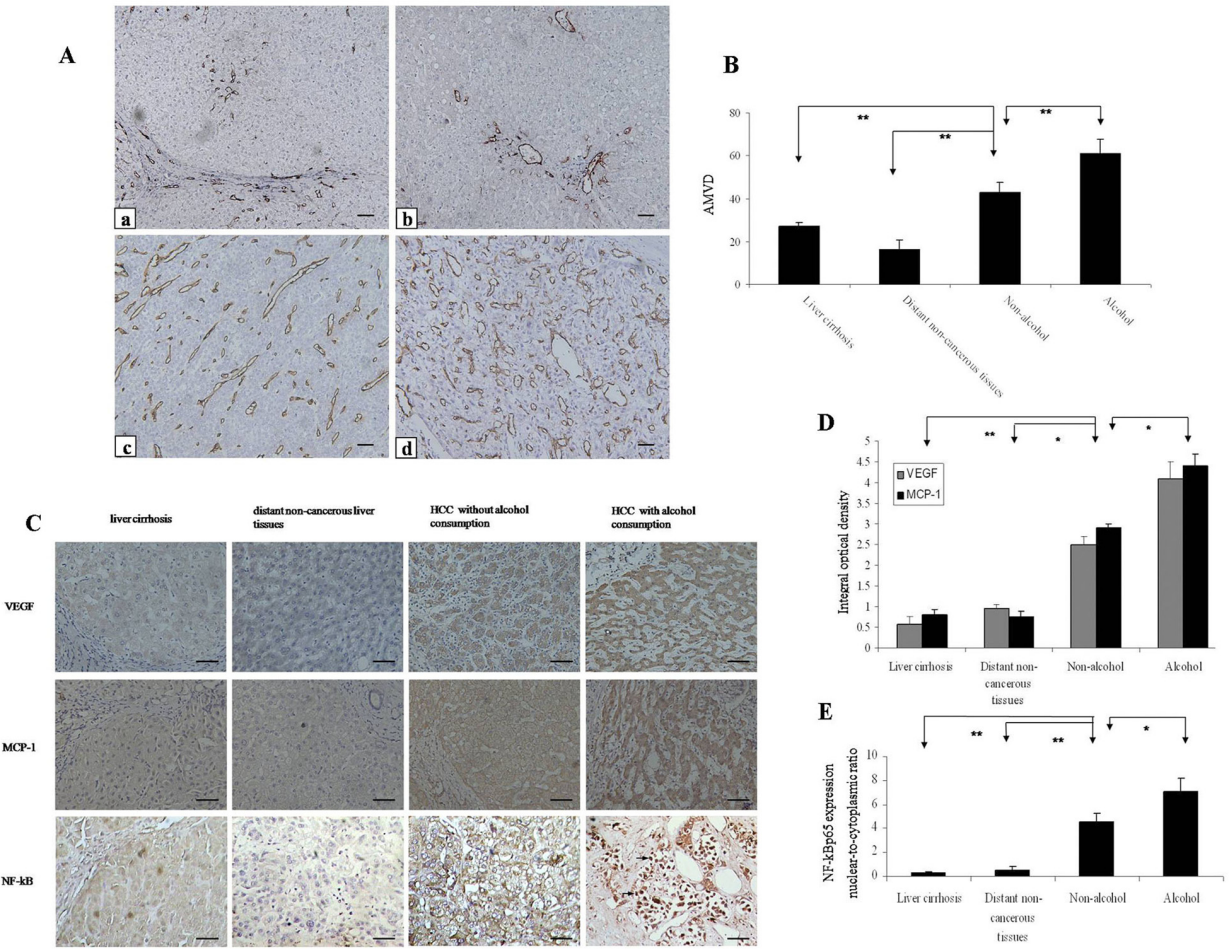
**Effects of chronic alcohol consumption on the expression of CD31, VEGF, MCP-1 and NF-kB in HCC patients. (A)** Immunohistochemical analysis of new blood vessels with anti-CD31 staining in different liver tissues. a: Liver cirrhosis; b: Distant non-cancerous liver tissues in HCC patients; c: HCC tissues of no-alcohol drinking patients; d: HCC tissues of alcohol-drinking patients; a-d: (Bar: 200 μm). The brown staining indicates the presence of microvessels. **(B)** Quantitative analysis of average microvessel density (AMVD, the number of microvessels per mm^2^ area) indicated that alcohol consumption significantly increased the number of new blood vessels (61.0 ± 6.78 in alcohol drinking patient *vs* 42.6 ± 4.82 in no-alcohol drinking patients, ** *P* < 0.01). **(C)** Immunohistochemical analysis of VEGF, MCP-1 and NF-κBp65 in liver tissues and HCC. (Bar: 100 μm). Arrows indicate NF-kBp65^+^ brown stain, which mainly found in the nuclei of tumor cells. **(D)** Quantitative analysis of VEGF, MCP-1 was determined by integral optical density. **(E)** Quantitative analysis of NF-κBp65 immunohistochemistry, as given by the nuclear-to-cytoplasmic ratio of NF-κBp65 positivity, n = 3.

To understand the underlying mechanisms, we first sought to determine whether alcohol consumption affected tumor angiogenesis. We compared average micrcovessel density (AMVD) in liver cirrhosis tissue, distant non-cancerous tissues, HCC tissues of nondrinkers and alcohol users. As showed in Figure 2A and B, AMVD in HCC tissue was higher than liver cirrhosis tissue and distant non-cancerous tissues, indicating higher angiogenesis in tumor (42.6 ± 4.82 *vs* 27.2 ± 1.48 and 42.6 ± 4.82 *vs* 16.2 ± 4.43, *P* < 0.01). More importantly, AMVD in HCC tissues of alcohol users was significantly higher than that of nondrinkers.

### Alcohol consumption is associated with higher expression of VEGF, MCP-1 and NF-κB in HCC tissues

To further understand the molecular mechanisms, we examined the expression of VEGF, MCP-1 and NF-ĸB in HCC tissues of nondrinkers and alcohol users. As shown in Figure 2C, VEGF and MCP-1 were expressed in tumor and hepatic cells. MCP-1 was also present in interstitial monocytes cells. Higher expression of VEGF and MCP-1 was observed in HCC tissues compared to cirrhosis and distant non-cancerous liver tissues (Figure 2D). Moreover, minimum NF-κB was mainly observed in the cytoplasm of hepatocytes in cirrhosis and distant non-cancerous liver tissues (Figure 2C and E), while in HCC tissues, strong expression was mainly located in the cytoplasm and nucleus of cancer cells. In all cases, the intensity for VEGF, MCP-1 and NF-κB immunostaining in the HCC tissues of alcohol users was much stronger than that of nondrinkers, even though tissues staining of drinkers and nondrinkers within the same TNM stage were compared.

### Involvement of reactive oxygen species (ROS) and NF-κB in ethanol-stimulated migration/invasion and tumor angiogenesis of HepG2 cells

**Figure 3:**
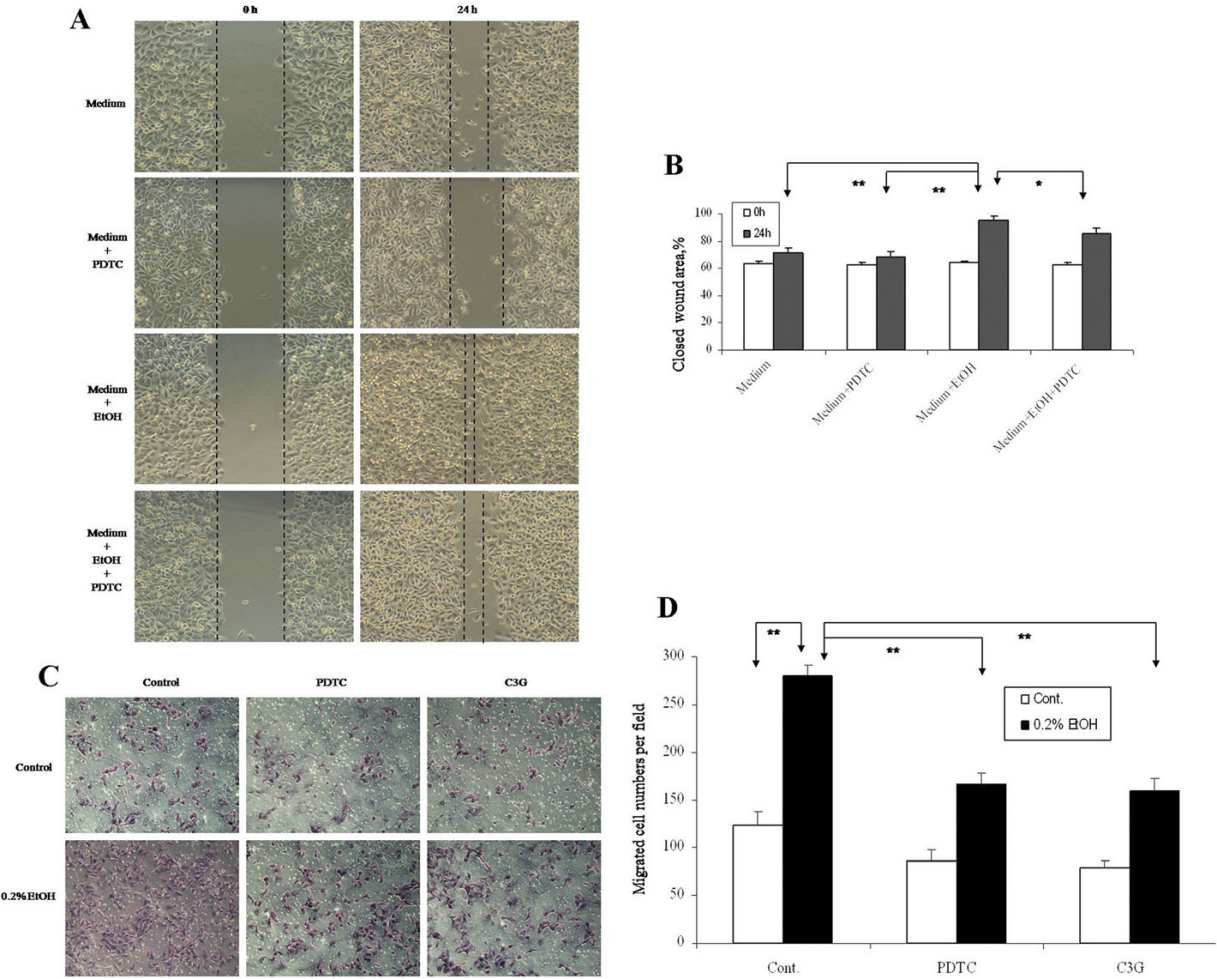
**Effects of ethanol on the migration/invasion of HepG2 cells** ***in vitro.*** **(A)** The migration of HepG2 cells was analyzed by the wound healing assay as described in the Materials and methods. HepG2 cells were exposed to ethanol (0.2%) with/without PDTC (20 μM) for 24 hours. Representative images of wound healing at 0 and 24 hours are shown. **(B)** Quantification of the migration of HepG2 cells. **(C)** The invasion of HepG2 cells was analyzed by a matrigel invasion assay. HepG2 cells were plated into the upper compartments of the matrigel invasion chambers and exposed to ethanol (0.2%) with/without PDTC (20 μM) or C3G (20 μM) for 48 hours. Images of cells migrating through the chambers were shown. **(D)** The cells migrated through the chamber were quantified. *P <0.05, **P <0.01, n = 3.

The clinical data from human studies indicated a positive association between alcohol consumption and enhanced progression and metastasis of HCC. We therefore sought to determine the causal relationship in experimental models. We first evaluated the effects of ethanol on the migration/invasion of human hepatocarcinoma HepG2 cells. Cell migration was evaluated by wound healing assay and cell invasion was determined with a Matrigel Boyden Chamber Assay. As shown in Figure 3A and B, ethanol exposure (0.2%) significantly increased the migration/invasion of HepG2 cells. The Matrigel assay indicated that ethanol exposure increased the invasion of HepG2 cells (Figure 3C and D, *P < 0.05, ** *P* < 0.01).

**Figure 4:**
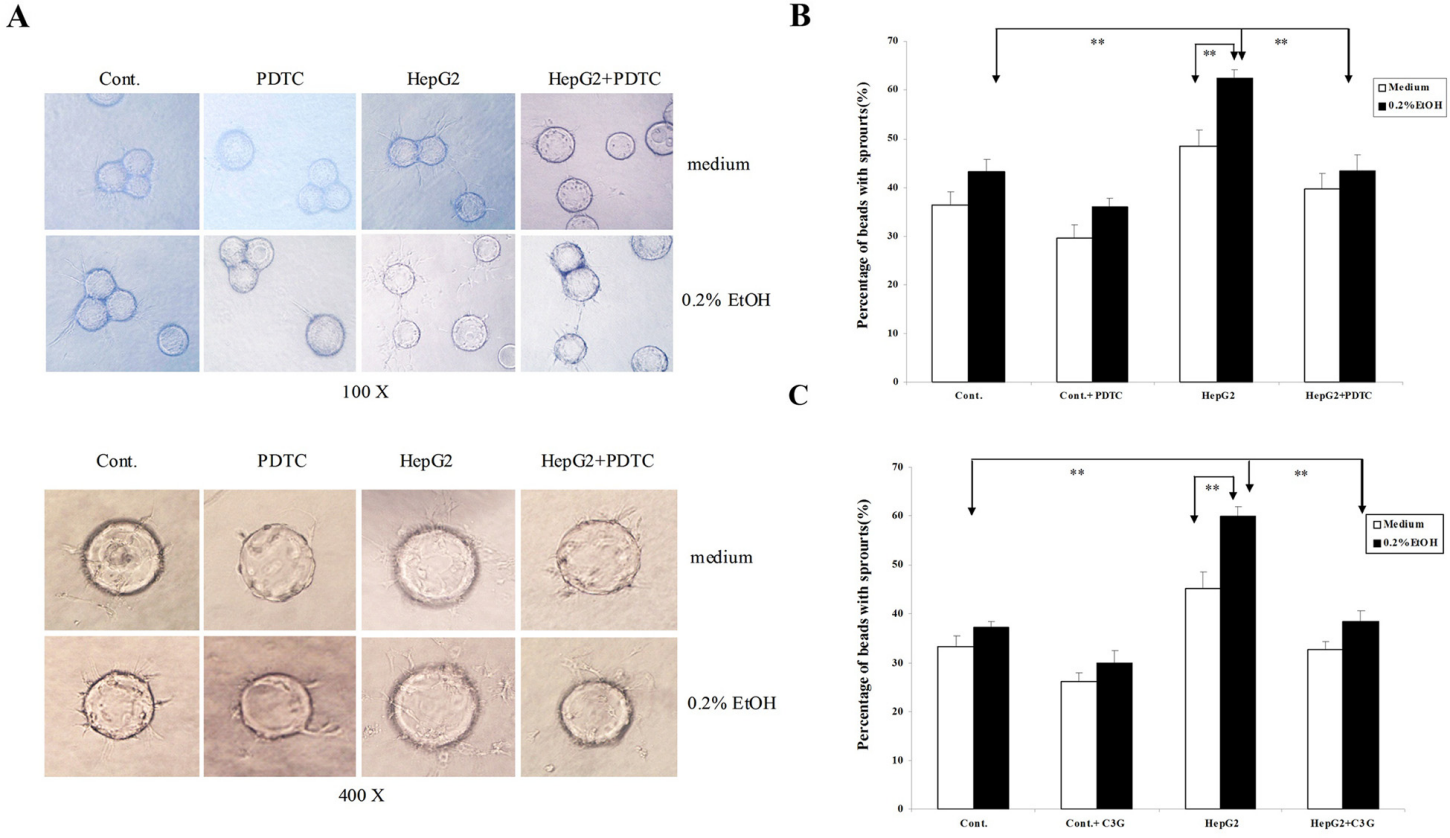
**Effects of ethanol on tumor angiogenesis in a 3-D model** ***in vitro.*** Tumor angiogenesis was analyzed using a 3-D co-culture system as described in Materials and methods. **(A)** Representative images of sprouts from HUVEC cells with bead under various culture conditions are shown. **(B)** HUVEC and HepG2 co-cultures were treated with ethanol (0.2%) with/without PDTC (20 μM). The angiogenesis which was indicated by the growth of endothelial sprouts was measured. **(C)** HUVEC and HepG2 co-cultures were treated with ethanol (0.2%) with/without C3G (20 μM). The angiogenesis which was indicated by the growth of endothelial sprouts was measured. **P < 0.01, n = 3.

**Figure 5:**
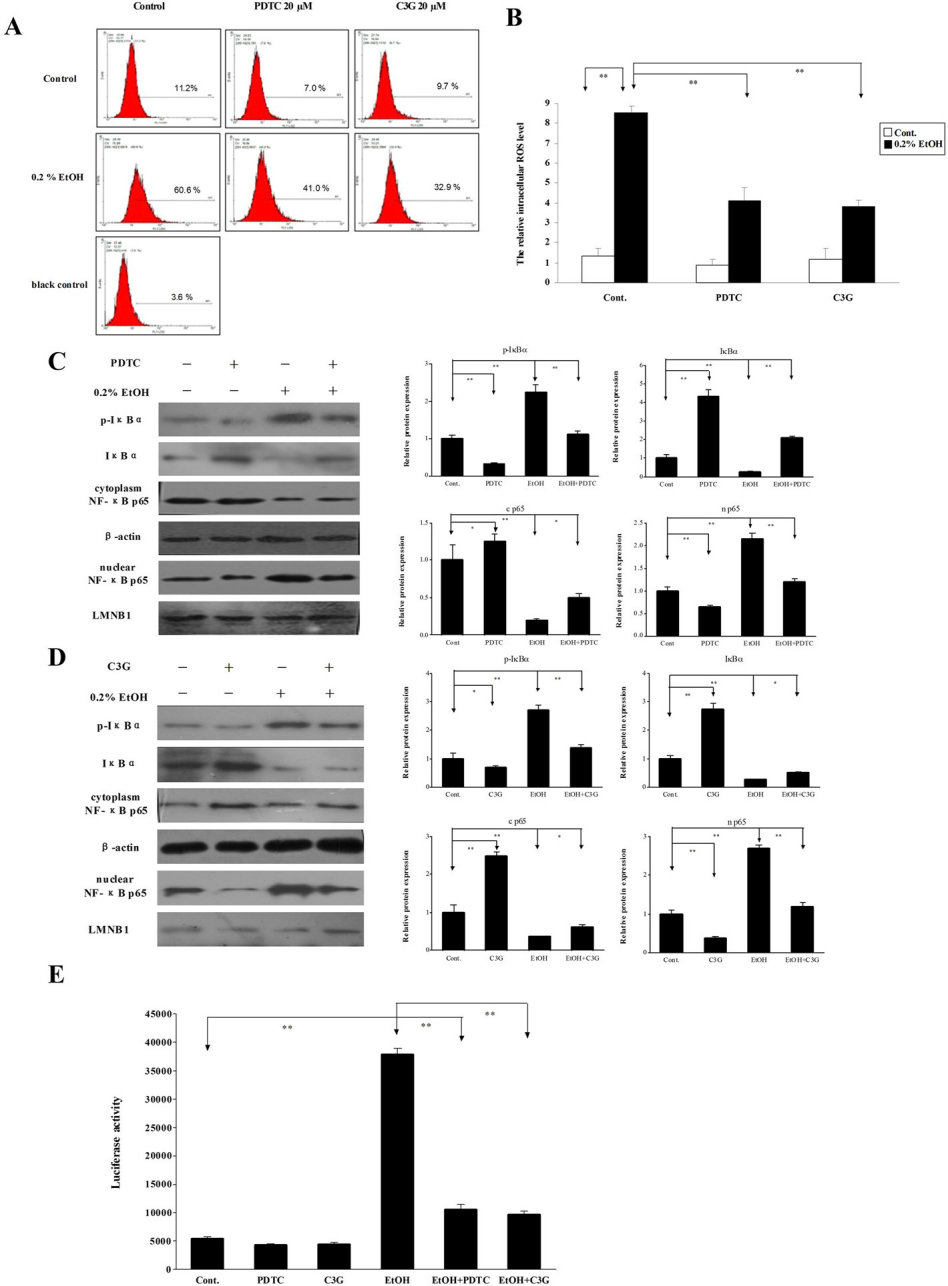
**Effects of ethanol on intracellular ROS and the activation of NF-kB. (A)** HepG2 cells were treated with ethanol (0.2%) with/without PTDC or C3G for 0.5 hours. Intracellular ROS was detected by flow cytometry as described in Materials and methods. **(B)** The relative levels of intracellular ROS were quantified. **P < 0.01. **(C-D)** HepG2 cells were treated with ethanol (0.2%) with/without PTDC or C3G for 2 hours. Expression of IkBα, p-IkBα and NF-kB p65 in cytoplasm and nuclei was analyzed by immunoblotting (left panel). The relative expression levels were quantified with a densitometry (right panel). *P < 0.05, **P < 0.01. **(E)** NF-kB activity was detected by the luciferase reporter gene assay. **P < 0.01, n = 3.

We next used a 3D co-culture model to evaluate tumor angiogenesis. In this system, human umbilical vein endothelial cells (HUVEC) attached to Cytodex beads are able to form a 3D capillary tube-like network (sprouts), indicative of angiogenesis. As shown in Figure 4A-C, the beads with sprouts were 36.4% in HUVEC alone group and 48.7% in HUVEC co-cultured with HepG2 cells. Ethanol exposure (0.2%) significantly increased the percentage of beads with sprouts (61.1%) in HUVEC/ HepG2 cell co-culture. These results indicated that ethanol stimulated tumor angiogenesis. The effects of ethanol were inhibited by PDTC or C3G. PDTC is a known NF-κB inhibitor and a metal chelator with some antioxidant property, while C3G inhibits ROS and mitigate ethanol-induced oxidative stress. However, the MTT assay showed that C3G and PDTC (20 μM) did not affect the proliferation of HepG2 cells (data not shown). To test the involvement of ROS and NF-κB signaling in this process, we demonstrated that ethanol induced intracellular ROS accumulation and C3G and PDTC inhibited ethanol-stimulated ROS production in HepG2 cells (Figure 5A and B). Since NF-κB is also an important redox-sensitive transcription factor and ethanol increased intracellular ROS level, we postulated that ethanol might activate NF-κB signaling. In the canonical NF-κB pathway, NF-κB activation depends on IκBα phosphorylation and degradation. We thus examined the effect of ethanol on the expression of active p65 NF-κB protein in HepG2 cells. As show in Figure 5C-5E, ethanol reduced cytoplasmic p65 NF-κB while increased nuclear p65 NF-κB, indicating that ethanol stimulated nuclear translocation of p65 NF-κB. Ethanol also enhanced IκB-α phosphorylation and decreased the levels of IκB-α. These results indicated that ethanol exposure activated NF-κB signaling. Consistently, PDTC reduced IκBα phosphorylation and p65 NF-κB nuclear translocation, while increased levels of IκB-α. Similarly, C3G also abolished ethanol-induced activation NF-κB, suggesting that ethanol-activated NF-κB signaling was mediated by ROS.

We further examined the effects of ethanol on the transcription of NF-κB using a luciferase reporter. As shown in Figure 5E, ethanol increased NF-κB transcriptional activity, while C3G or PDTC inhibited ethanol-stimulated NF-κB transcriptional activity. Taken together, ethanol may stimulate the NF-κB pathway by inducing ROS production, which in turn increases migration/invasion and tumor angiogenesis.

### Alcohol enhances liver tumor growth and aggressiveness in mice

**Figure 6:**
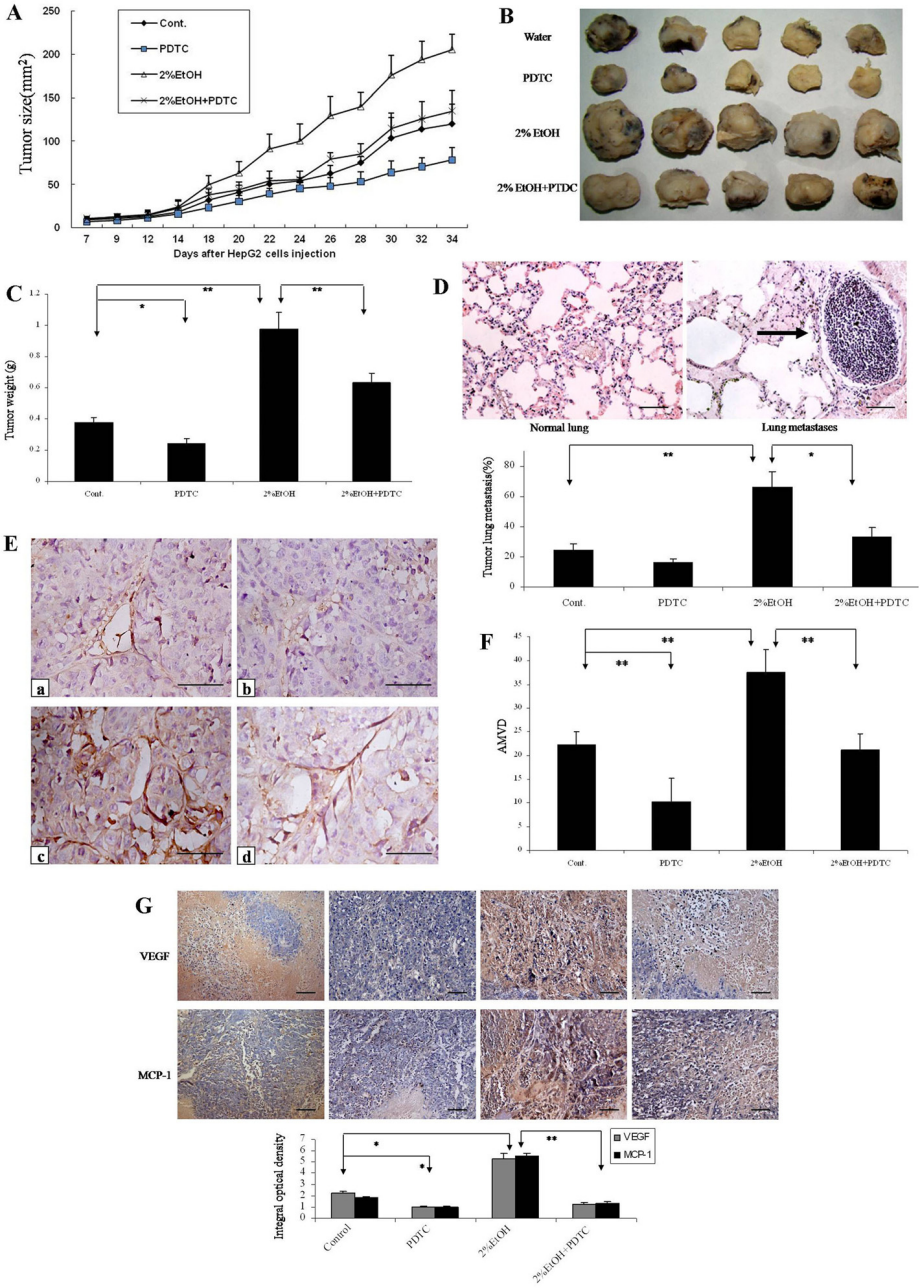
**Effects of ethanol on tumor development in a xenograft model. (A)** HepG2 cells were implanted subcutaneously in nude mice. The mice were exposed to ethanol in drinking water (0 or 2% ethanol). Mice were injected with PDTC (0 or 100 mg/kg). The size of tumor was measured every other day. **(B)** At the end of experiments, tumors were removed for subsequent analyses. A representative image shows tumors from control and ethanol-exposed mice with/without PDTC treatment. **(C)** Tumor weight was determined and presented as the means ± SD (n = 12). **(D)** Tumor metastases in the lungs were analyzed. A representative microphotograph shows metastatic carcinoma nodes in the lungs of ethanol-exposed mice (arrow, top panel; Bar: 100 μm). The percentage of mice which were positive for lung metastasis was quantified (bottom panel). **(E)** Microvessels in tumor tissues were detected by CD31 immunohistochemistry (Bar: 50 μm). a: water control, b: water plus PDTC, c: 2% ethanol, d: 2% ethanol plus PDTC. **(F)** AVMD was determined as described in Figure 2. **(G)** VEGF and MCP-1 in tumor tissues were detected by IHC (Bar: 100 μm). *P < 0.05, **P < 0.01, n = 3.

We sought to further confirm our findings in a mouse xenograft model in which HepG2 cells were implanted subcutaneously in nude mice. As shown in Figure 6A-C, ethanol exposure significantly enhanced the rate of tumor growth; the size/weight of tumor in ethanol-exposed mice was significantly larger than that in control groups. Ethanol exposure also significantly increased tumor metastasis (Figure 6D), angiogenesis (Figure 6E) and the expression of VEGF and MCP-1 (Figure 6G).

In addition, PDTC treatment significantly inhibited ethanol stimulation of tumor growth and metastasis in mice (Figure 6A-D). Consistent with the observation made in the *in vitro* model, PDTC inhibited ethanol-promoted angiogenesis (Figure 6E and F). PDTC also reduced the basal expression VEGF and MCP-1 and blocked ethanol-induced increase in VEGF and MCP-1 expression in liver tumors (Figure 6G).

## Discussion

The association between alcohol consumption and the risk of HCC has been well-established. For example, a study with a cohort of 19,000 drinkers confirmed that alcohol enhances the risk of upper gastrointestinal tumors including liver cancer [16]. Rehm et al. [17] reported that RRs of liver cancer for low (0–39.99 g/d), hazardous (40–59.99 g/d) and harmful (60+ g/d) levels of alcohol consumption compared to never drinkers are 1.45, 3.03, and 3.60 respectively. Hennig et al. suggested the close association between alcohol consumption and HCC progression *in vitro* [18]. Huang et al. also confirmed that ethanol consumption induces hepatocellular carcinoma cell metastasis by changing the extracellular matrix *in vitro* and this ability was reduced by food components such as pterostilbene and curcumin analogues [19]. In a mouse model of hepatocaricinogenesis, Brandon-Warner et al. found that chronic ethanol feeding accelerated hepatocellular carcinoma progression in male mice [20]. In our study, we demonstrate a positive correlation between alcohol consumption and enhanced HCC progression, even though the cohort of this study is relatively small (82 patients). Donato et al. showed that heavy alcohol consumption (>80 g of ethanol per day) is associated with the increased risk of HCC compared to non-drinkers [21]. However, our data indicated that the moderate and heavy drinking (35–87.5 g/day and ≥ 87.5 g/day) was associated with enhanced HCC progression. There are many factors underlying this discrepancy. A larger cohort may be needed to provide more definite conclusion. In our cohort, the number of male patients is much larger than that of female; this is consistent with the epidemiological findings that males are about two to four times more likely to develop HCC than females [22]. In future study, analysis of a larger cohort may be necessary to evaluate the interaction between alcohol consumption and other risk factors, such as Hepatitis B virus or hepatitis C virus which are known risk factors for the development of liver cancer due to chronic liver inflammation [23]. Nevertheless, our clinical data provides the necessary foundation for our further experimental studies.

HCC is a vascular-dependent malignant tumor; an increase in tumor dimension above 0.5 mm will induce the proliferation of vascular endothelial cells and angiogenesis which is essential for tumor growth and metastatic dissemination [24]. Our previous study and results from others showed that alcohol may promote tumor angiogenesis in animals [13,25,26]. The current study for the first time demonstrates that alcohol consumption is closely associated with a higher micrcovessel density in HCC tissues, which implied that alcohol potentially enhances tumor angiogenesis in HCC patients. This interpretation is further supported by experimental data. For example, alcohol increases micrcovessel density in tumor tissues in a HCC xenograft model . Furthermore, we show that alcohol can stimulate tumor angiogenesis in a 3D model of tumor/endothelial cell co-culture system. Together, these results indicate that enhanced angiogenesis is a potential mechanism for alcohol-promoted progression of HCC.

VEGF and MCP-1 are key mediators for tumor angiogenesis and metastasis [27,28]. VEGF, as a pro-angiogenic factor, plays an essential role in the process of angiogenesis, including microvascular permeability, endothelial cell proliferation and tumor cell migration [29,30]. VEGF-positive tumors in HCC patients had much greater invasive potential and intrahepatic metastasis than VEGF-negative tumors [31]. Tan et al. [25] showed that alcohol increased the expression of VEGF in melanoma xenograft model. MCP-1 is also a potent pro-angiogenic chemokine which is associated with many malignant tumors progression by recruitment of macrophages and induction of angiogenesis [32,33]. In both ectopic and orthotropic xenograft models, the MVD was significantly increased in tumors over-expressing MCP-1 [34]. We have previously demonstrated that alcohol can stimulate the expression of MCP-1 in breast cancer cells, which caused an enhanced angiogenesis [13]. That a higher expression level of VEGF and MCP-1 is observed in HCC patients of alcohol-drinkers supports a role of VEGF and MCP-1 in alcohol promotion of HCC.

NF-κB plays a key role in tumorigenesis/progression and also regulates the expression of VEGF and MCP-1 [35]. Consistently, we show that the expression of NF-κB is higher in alcohol-drinkers HCC patients than in that in non-drinkers. Furthermore, PDTC, an inhibitor of NF-κB and an antioxidant, attenuates alcohol-induced VEGF and MCP-1 expression in mice, supporting that ethanol-stimulated expression of VEGF/MCP-1 is mediated by NF-κB signaling.

NF-κB activity is regulated by reactive oxygen species (ROS) [36]. We have previously shown that ethanol can induce intracellular ROS accumulation in breast cancer cells [37,38]. ROS plays an important role in carcinogenesis as a result of oxidative stress including oxidative injury, inflammation, and lipid peroxidation [39]. C3G, a potent antioxidant, has been shown to effectively inhibit ROS and mitigate ethanol-induced oxidative stress [38]. We confirm here that ethanol causes the accumulation of intracellular ROS in HepG2 cells and C3G scavenges ethanol-induced ROS. More importantly, C3G blocks ethanol-stimulated NF-κB promoter activities, suggesting that ethanol-induced ROS mediated NF-κB transcriptional activity. At cellular level, both PDTC and C3G effectively inhibit ethanol-induced migration/invasion of HepG2 cells and abolish the pro-angiogenic effect of ethanol in a 3-D model of tumor/endothelial cell co-culture. They also attenuate ethanol-induced tumor growth and metastasis in mice.

## Conclusions

**Figure 7:**
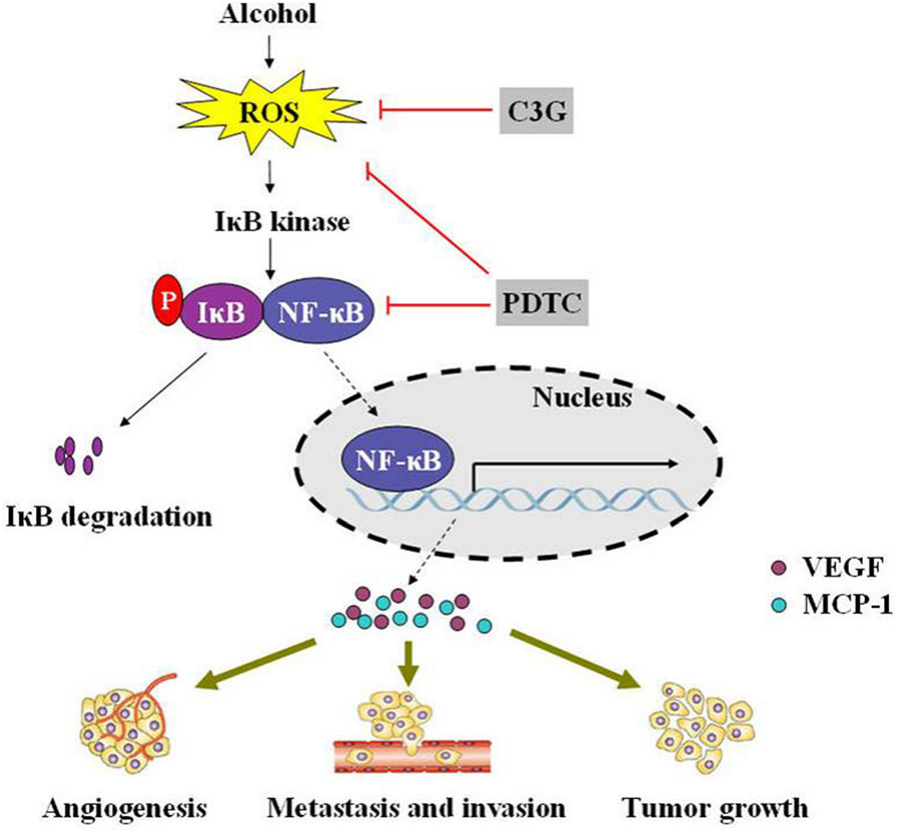
**Proposed working model for the role of the NF-kB signaling pathway in the regulation of ethanol-enhanced the progression of HCC.** (1) ROS induced by alcohol mediated activation of the NF-kB pathway. This effect can be abolished by C3G or PDTC. (2) Nuclear translocation of NF-kB via phosphorylation by the IkB kinase, which then binds target DNA that regulates VEGF and MCP-1 gene expression. Ultimately, the changes stimulated by alcohol enhance angiogenesis, tumor growth and metastasis.

In conclusion, our results indicate that alcohol consumption induces intracellular ROS accumulation which results in the activation of NF-κB and up-regulation of VEGF and MCP-1. VEGF and MCP-1 promotes tumor angiogenesis which may mediate ethanol-stimulated progression/metastasis of HCC (Figure 7).

## Materials and methods

### Drugs and reagents

Ethanol, fibrinogen, aprotinin, thrombin and Pyrrolidine dithiocarbamate (PDTC) were purchased from Sigma Chemical Co. (St. Louis, MO). Cyanidin-3-glucoside (C3G) was prepared as previously described [38]. Anti-MCP1 and anti-VEGF antibodies were obtained from Abcam (Cambridge, MA). Anti-NF-κB p65, IκBα, p-IκBα and anti-LMNB1 antibodies were purchased from Protein Tech Group (Chicago, IL, USA). Anti-β-actin was obtained from Cell signaling Technology (Danvers, MA). Anti-CD31 antibody was obtained from BD Pharmingen (San Diego, CA). Reactive oxygen species detection reagents were obtained from Invitrogen Molecular Probes (Eugene, OR). MTT assay kit was purchased from Roche Molecular Biochemicals (Indianapolis, IN). Matrigel Invasion Chambers were purchased from BD Biosciences (Bedford, MA). Cytodex 3 beads were purchased from Amersham Pharmacia Biotech (Piscataway, NJ).

### Clinical patient data

The medical records of 52 HCC patients, admitted to first hospital of Anhui Medical University, were retrospectively analyzed between January and December 2009 (Table 1). Patients were asymptomatic at admission and diagnosed by examining the liver with computed tomography. All patients were performed anatomical liver resection. HCC and cirrhosis was confirmed by pathology. Cirrhosis developed in 76.9% (39 of 52) of patients. Forty-three patients (82.7%) were infected with hepatitis B virus. A series of demographic and clinical data were collected including alcohol consumption, tumor characteristics and pathologic stages. Survival and follow-up data were acquired by telephone interviews. With the approval of the first affiliated hospital of Anhui Medical University of Human Studies Committee, formalin-fixed paraffin-embedded tumor materials of 52 HCC patients were obtained. The histological sections of all cases were evaluated by oncopathologists.

### Cell culture and ethanol exposure

Human hepatoma cell line HepG2 and mouse embryonic fibroblast cell line NIH3T3 were provided by the Institute of Cell and Biochemistry, Chinese Academy of Sciences (Shanghai, China) and maintained in Dulbecco’s modified Eagle’s medium(DMEM) containing 10% fetal bovine serum (FBS), penicillin (100 U/ml)/streptomycin (100 U/ml), at 37°C with 5% CO2. The original primary human umbilical vein endothelial cells (HUVECs) were isolated from human umbilical veins by using collagenase (Roche Diagnostics, Switzerland) as previously described [40], and maintained on 0.2% gelatin coated in EGM-2 medium (Lonza, Walkersville, MD, USA) which consists of endothelial cell basal medium (EBM-2) with 10% FBS and other additives. Alcohol was added to the medium at concentrations of 0.2% v/v ethanol. Cell culture plates were placed on a rack inside a plastic container sealed with a tight-fitting lid. At the bottom of each container, there was a 200-ml water bath, which contained the same concentration of ethanol as in the cultural media. The containers were placed in a humidified environment and maintained at 37°C with 5% CO2.

### Animals and ethanol administration

Male nude mice (4–5 weeks old, 15–20 g, n = 12/group) were purchased from the Animal Center, Chinese Academy of Sciences. The ethanol was administrated in drinking water as described previously [13]. Briefly, mice were given 2% ethanol in drinking water for a 12 h period during the night and then replaced with water without ethanol at day time. The mice in the control group were provided with regular drinking water only. All procedures were carried out according to the guidelines of the Animal Welfare Act approved by the Institutional Animal Care and Use Committee of Anhui Medical University. The blood ethanol concentration (BEC) was determined at 6:00 a.m. using an Analox AM1 Alcohol Analyzer (Analox Instruments, Lunenburg, MA) as previously described [41]. The BEC was 56.18 ± 11.6 mg/dl.

### Mouse model of tumor xenograft

HepG2 human hepatoma cells were implanted subcutaneously in nude mice according to the previous study [13]. Briefly, three days after ethanol exposure, HepG2 cells (1 × 10^6^ in 100 μl PBS) were injected subcutaneously into mice using a 23-gauge needle. The mice were continually provided with 2% ethanol in drinking water or regular drinking water without ethanol. The size of the tumors was monitored every 2 days; two perpendicular dimensions of tumors were measured with a dial caliper. The volume was calculated based on the formula: V = 0.25 a × b ^2^; a is the longest and b is the shortest dimension. At the end of experiment, animals were killed and the tumors were removed. Some of the tumor tissues were fixed with 10% neutral formalin for and immunohistochemical studies. To evaluate the effect of PDTC on ethanol-mediated tumor growth, PDTC [100 mg/kg in 100 μl of Dimethyl sulfoxide (DMSO)] or DMSO alone was injected intraperitoneally 2 days following ethanol exposure. PDTC was administered every three days. The dosage of PDTC was selected based on previous studies [42]. There were seven mice for each treatment group.

### Histology and immunohistochemistry

Immunohistochemical (IHC) procedure was performed generally as described [43]. Briefly, Five-micrometer-thick sections were cut and deparaffinaged in xylene prior to rehydration using gradient alcohol. For antigen retrieval, sections were treated with citrate buffer saline (pH 6.0) for 15 minutes at 95°C in a microwave oven. After blocking with 10% normal goat serum for 30 minutes at room temperature, the sections were incubated with primary antibodies: anti-MCP-1 (1:100), anti-VEGF (1:150), anti-NF-κBp65 (1:100), or anti-CD31 (1:100) overnight at 4°C. Following incubation, sections were washed with phosphate buffered solution and incubated with horseradish peroxidase-conjugated goat anti-rabbit IgG for 1 h at room temperature. The avidin-biotin complex was added and the enzyme activity was visualized with 3, 3’-diaminobenzidine (DAB). Negative controls were prepared using PBS instead of the primary antibody. The average microvessel density (AMVD) of ten selected microscopic fields was calculated and expressed as the number of microvessels per mm^2^ area, according to previous report [44]. The integral optical density (IOD) of the stained sections was measured with a computer-assisted image-processing and analytical system. NF-κBp65 expression was analyzed by counting NF-κBp65-positive hepatocytes within 50 HPF with discrimination of the intracellular distribution serving as an indicator of NF-κB nuclear translocation. The nuclear-to-cytoplasmic ratio was calculated by dividing the number of cells with nuclear NF-κBp65 positivity by the number of cytoplasmic NF-κBp65 positive cells.

### Analysis of experimental lung metastasis

After 5 weeks of ethanol exposure, mice in four groups (n = 22 for each group) were sacrificed. The fresh lung samples were harvested and fixed with 10% formalin for histopathology analysis. Tissues were paraffin-embedded and sectioned at a thickness of 5 μm. The sections were stained with hematoxylin–eosin (H&E) and then examined under the microscope to evaluate the morphological changes of metastasis tumors.

### Detection of intracellular reactive oxygen species

Intracellular reactive oxygen species (ROS) levels were measured using the fluorescent dye CM-H2DCFDA (Invitrogen Corporation, Carlsbad, CA) as previously described [45]. Briefly, HepG2 cells were trypsinized and seeded onto 6-well plates pretreated with/without C3G (20 μM) or PDTC (20 μM) for 0.5 hours before ethanol exposure. After the treatment, cells were washed with cold PBS and incubated with 5 μM CM-H2DCFDA for 30 min, followed by several additional washes with cold PBS. Cells were trypsinized and transferred into polystyrene round-bottom tubes; intracellular ROS levels were measured with a flow cytometer (FACScalibur, BD Biosciences, San Jose, CA) at an emission wavelength of 525 nm.

### Preparation of protein extracts

Cells (5 × 10^6^) were harvested, resuspended in 50 μl of lysis buffer A (10 mM Hepes, 10 mM KCl, 0.1 mM MgCl2, 0.1 mM EDTA, 2 μg/ml leupeptin, 2 μg/ml pepstatin, 0.5 mM PMSF, pH 7.9), incubated on ice for 10 min and centrifuged for 10 min at 800 × g at 4°C. The supernatant was treated as the cytoplasmic fraction. The pellet (nuclei) was washed with buffer A and nuclear proteins were extracted in presence of 50 μl of buffer B (10 mM Hepes, 400 mM NaCl, 1.5 mM MgCl2, 0.1 mM EDTA, 2 μg/ml leupeptin, 2 μg/ml pepstatin, 0.5 mM PMSF, pH 7.9). Protein concentration was determined using a BCA protein assay kit (Pierce, Rockford, IL) according to the manufacturer's instructions.

### Western blotting analysis

SDS–PAGE and Western blotting were performed according to standard procedures. Briefly, protein samples (10 μg) were subjected to electrophoresis on 4-12% SDS-polyacrylamide gradient gels and transferred to a PVDF membrane. After overnight blocking with 2% BSA in TBS-Tween 0.1%, the membranes were probed with anti-NF-κB p65 (1:1000), IκBα (1:500), p-IκBα (1:1000), β-actin (1:1000) or anti-LMNB1 (1:1000) antibodies overnight at 4°C. Anti-rabbit HPR conjugated antibody was used as a secondary antibody followed by enhanced chemiluminescence reaction. All Western blotting were repeated three times.

### Luciferase reporter gene assay

NIH3T3 cells were trypsinized and seeded in 96-well plates at an initial density of 2 × 10^4^ cells/well in DMEM containing 10% FBS at 37°C with 5% CO2 overnight. The cells at 50%–80% confluence were performed with 6 μg of pGL4.32 [luc2P/NF-κB-RE/Hygro] plasmid (Promega) transfected with Lipofectamine™ 2000 Transfection Reagent (Invitrogen) for normalizing transfection efficiency. The non-transfected cells were used as blank group. At 48 hours after transfection, cells were collected and resuspended in passive lysis buffer (Promega). Then the cells were exposed with/without ethanol in the presence or absence of PDTC or C3G. After incubation for 2 h, Luciferase activity was evaluated with a dual luciferase assay system (GloMax 96 Microplate Luminometer, Promega) according to manufacturers.

### Cell proliferation assay

The MTT assay was employed to determine the number of viable cells in culture. Briefly, the cells were plated into 96-well plates and exposed to ethanol with/without C3G (20 μM) or PDTC (20 μM) for indicated times. After the treatment, 10 μl of MTT reagent was added into each well and the plates were incubated at 37°C for 4 h. After lightly vortexing the plate on an orbital shaker, the spectrophotometric absorbance was read on a microplate reader (Bio Rad Model 3550-UV) at 595 nm. Each individual experiment was performed at least three times.

### Wound healing migration assay

Wound healing assay was carried out to determine the cell migration ability of tumor cells. The wound healing migration assay was performed as described previously [46]. Briefly, HepG2 cells were plated on 6-well plates and grown to 80% - 90% confluence. Sterilized one-milliliter pipette tip was used to generate a wound across the cell monolayer, and the plates were washed with PBS. The cells were then treated with ethanol (0.2% v/v) in the presence or absence of PDTC (20 μM) for 24 h. Cells migrated into the wounded area or protruded from the border of the wound were visualized and photographed under the inverted microscope (Olympus, Japan).

### Cell invasion assay

Cell invasion was performed in BD Bio Coat Matrigel Invasion chambers (BD Biosciences, Clontech; Bedford, MA, USA) with a porous polycarbonate membrane (8.0 micron pore size) as previously described [47]. Briefly, HepG2 cells (5 × 10^5^) were seeded onto upper chambers and treated with ethanol in the presence or absence of C3G or PDTC(20 μM). The lower compartment of the chamber was filled with medium containing 2%BSA served as chemoattractants for the cells. The chambers were cultured at 37°C in 5% CO2 for 48 h to allow tumor cell invasion through the matrix. Migrated cells were fixed with 100% methanol and stained with Giemsa for invasion assay. The filters were photographed and the cells were counted. The invasion assay was repeated in five separate experiments, with control and experimental groups performed in parallel.

### Three-dimensional (3D) endothelial cell and tumor co-culture system

3-D model of endothelial cell and tumor cell co-culture system was performed to investigate the effect of ethanol on tumor angiogenesis as described previously [13]. In this model, endothelial cells were induced to form a 3D capillary tube-like network on a fibrin gel bead system in the presence or absence of tumor cells. In brief, HUVEC or HepG2 cells (1 × 10^6^) were trypsinized and mixed with Cytodex beads (3 × 10^3^) in DMEM medium. After incubation, the mixtures of cells/Cytodex beads were re-suspended in medium containing 2.5 mg/ml fibrinogen and 0.15 U/ml aprotinin. A volume of 0.5 ml fibrinogen/bead solution was added to 24-well cell culture plates that were pre-coated with 0.625 U of thrombin. After coagulation, the resulting fibrin gels contained endothelial cells (HUVEC or HepG2) adhering to the beads. For co-culture of endothelial cells/tumor cells, the medium collected from HepG2 cells with/without ethanol in presence or absence of C3G or PDTC were added on top of the fibrin gels. The culture media was changed every two days. At different time points, cell morphogenesis was observed using an inverted phase-contrast microscope. Tube formation was quantified by counting branches from four representative fields per replicate. These experiments were performed in triplicate.

### Statistical analysis

Data were presented as means ± standard deviations and analyzed by ANOVA, followed by Student-Newman-Keuls test. The correlations between alcohol consumption and clinicopathological features were analyzed by Pearson Chi-square test. Multivariable binary logistic regression was carried out to estimate odds ratio (OR) with its 95% confidence intervals (CI) in order to assess the risk factors for HCC aggressiveness. The multivariate Cox proportional hazards regression model was employed to estimate the prognostic factors associated with HCC recurrence. For the survival analysis after surgical resection, the data were processed using the Kaplan-Meier method and were compared using the log-rank test. Statistical analysis was conducted with SPSS 16.0 (IBM Corporation, Chicago, IL, United States). A value of *P* < 0.05 was considered statistically significant.

## Abbreviations

HCC: Hepatocellular carcinoma
HepG2: Human hepatoma cell line
PDTC: Pyrrolidine dithiocarbamate
C3G: Cyanidin-3-glucoside
IHC: Immunohistochemistry
VEGF: Vascular endothelial growth factor MCP-1, Monocyte chemotactic protein-1
ROS: Reactive oxygen species
NF-κB: Nuclear factor kappa B
IkBα: Inhibitor of NF-kB
p- IkBα: Phosphorylation of IkBα
TNM: Classification of malignant tumours
HUVECs: Human umbilical vein endothelial cells
NIH3T3: Mouse embryonic fibroblast cell line
BEC: Blood ethanol concentration
AMVD: Average microvessel density
OR: Odds ratio
RRs: Relative risks
CI: Confidence interval
H&E: Hematoxylin–eosin
