# Editorial expression of concern: Activation of the NF-κB pathway as a mechanism of alcohol enhanced progression and metastasis of human hepatocellular carcinoma

**DOI:** 10.1186/s12943-022-01693-8

**Published:** 2022-12-15

**Authors:** Fei Wang, Jin-Lian Yang, Ke-ke Yu, Mei Xu, You-zhi Xu, Li Chen, Yan-min Lu, Hao-shu Fang, Xin-yi Wang, Zhong-qian Hu, Fei-fei Li, Lixin Kan, Jia Luo, Si-Ying Wang

**Affiliations:** 1 Department of Pathophysiology, School of Basic Medical Science, Anhui Medical University, 81 MeiShan Road, Hefei, Anhui 230032 P.R. China; 2 Department of Burns, The First Affiliated Hospital of Anhui Medical University, Hefei, Anhui 230022 P.R. China; 3 Department of Pharmacology and Nutritional Sciences, University of Kentucky College of Medicine, Lexington, Kentucky 40536 USA; 4 Department of Clinical Medicine, Anhui Medical University, Hefei, Anhui 230032 P.R. China

**Correction: Mol Cancer 14, 10 (2015)**

**https://doi.org/10.1186/s12943-014-0274-0; Published: 27 January 2015**

The Editor-in-Chief is issuing an editorial expression of concern to alert readers that this article shows signs of irregularities within the 1st and 4th image of VEGF in Figure 6G after this was highlighted by a reader. The Editor-in-Chief advises the readers to interpret the data with due caution. All authors agree to this Editorial Expression of Concern.

